# Frequency of Eating in the US Population: A Narrative Review of the 2020 Dietary Guidelines Advisory Committee Report

**DOI:** 10.1093/cdn/nzac132

**Published:** 2022-08-29

**Authors:** Regan L Bailey, Heather J Leidy, Richard D Mattes, Steven B Heymsfield, Carol J Boushey, Namanjeet Ahluwalia, Alexandra E Cowan, TusaRebecca Pannucci, Alanna J Moshfegh, Joseph D Goldman, Donna G Rhodes, Eve E Stoody, Janet de Jesus, Kellie O Casavale

**Affiliations:** Institute for Advancing Health through Agriculture, Texas A&M University, College Station, TX, USA; Departments of Nutritional Sciences and Pediatrics, Dell Medical School, University of Texas at Austin, Austin, TX, USA; Institute for Advancing Health through Agriculture, Texas A&M University, College Station, TX, USA; Pennington Biomedical Research Center, Louisiana State University, Baton Rouge, LA, USA; Epidemiology Program, University of Hawaii Cancer Center, Honolulu, HI, USA; Division of Health and Nutrition Examination Surveys, National Center for Health Statistics, Centers for Disease Control and Prevention, Hyattsville, MD, USA; Institute for Advancing Health through Agriculture, Texas A&M University, College Station, TX, USA; Center for Nutrition Policy and Promotion, Food and Nutrition Services, US Department of Agriculture, Alexandria, VA, USA; Agricultural Research Service, US Department of Agriculture, Beltsville, MD, USA; Agricultural Research Service, US Department of Agriculture, Beltsville, MD, USA; Agricultural Research Service, US Department of Agriculture, Beltsville, MD, USA; Center for Nutrition Policy and Promotion, Food and Nutrition Services, US Department of Agriculture, Alexandria, VA, USA; Office of Disease Prevention and Health Promotion, US Department of Health and Human Services, Rockville, MD, USA; Office of Nutrition and Food Labeling, Center for Food Safety and Applies Nutrition, Food and Drug Administration, US Department of Health and Human Services, College Park, MD, USA

**Keywords:** frequency of eating, meal patterns, dietary quality, NHANES, eating behaviors

## Abstract

**Background:**

A person's daily nutrient intake and overall nutritional status are determined by a complex interplay of the types and amounts of foods ingested in combination with the timing and frequency of eating.

**Objectives:**

The aim was to summarize frequency of eating occasion data examined by the 2020 Dietary Guidelines Advisory Committee, the macronutrient contributions they provide, and meal frequency relative to dietary quality among the US population (≥2 y), with a focus on sex, age, race/Hispanic origin, and income.

**Methods:**

Demographic and 24-h recall data from the 2013–2016 NHANES were examined. An eating occasion was defined as “any ingestive event (e.g., solid food, beverage, water) that is either energy yielding or non-energy yielding”; all eating occasions were further divided into discrete meals and snacks. Frequency of meals and snacks was defined as “the number of daily EOs [eating occasions],” respectively. Diet quality was assessed via the Healthy Eating Index (HEI)–2015.

**Results:**

Most Americans consume 2 (28%) to 3 (64%) meals on a given day and >90% consume 2 to 3 snacks on that day. Adult, Hispanic, and non-Hispanic Black and lower-income (<131% family poverty-to-income ratio) Americans had a lower frequency of eating than children or adolescents, non-Hispanic White, and non-Hispanic Asian Americans and higher-income Americans, respectively. Americans who reported 3 meals on a given day consumed a diet higher in dietary quality than Americans who consumed 2 meals on a given day (HEI-2015: 61.0 vs. 55.0), regardless of population subgroup.

**Conclusions:**

The frequency of the types of eating occasions differs according to age, race and Hispanic origin, and income. Dietary quality is associated with the number of meals consumed. Healthy dietary patterns can be constructed in a variety of ways to suit different life stages, cultural practices, and income levels; improved diet quality and careful consideration of nutrient density when planning meals are warranted.

## Introduction

Eating is a human behavior critical for meeting energy and nutrient needs and for sustaining growth and development, metabolic function, and maintenance. Eating behaviors can either promote or impair a healthy lifestyle and can therefore strongly influence the quality and length of one's life (1, 2). While eating behaviors are commonly referred to in terms of the types and amounts of foods ingested, the timing and frequency of eating are also important factors. The daily nutrient intake and overall nutritional status of an individual is determined via a complex interplay of these 3 factors (3–6). The timing and frequency of eating may impact the types and/or amounts of foods eaten and may alter metabolic processes and responses (7–9). Thus, changes in frequency of eating could give rise to fluctuations in a person's nutritional status, as well as risk of some cardiometabolic diseases (7–9).

A wide variety of eating patterns exist in America, especially in terms of the frequency and timing of eating. The conventional American diet typically consists of approximately 3 meals per day (i.e., breakfast, lunch, and dinner). This 3-meal frequency of eating pattern is deeply rooted in American culture (10); however, a recent study designed to assess the frequency of eating, timing of eating occasions, and the labels applied to those eating occasions by Americans suggested that Americans now, on average, report over 5 eating occasions (i.e., meals or snacks) on a given day (10). Most eating occasions tend to cluster around specific times that coincide with the conventional naming of meals (11); however, several advantages and disadvantages are associated with this approach, given the broad variability in eating behaviors between different population subgroups in the United States.

This increase in the frequency of eating among the American population is directly related to an increase in snacking, although a lack of consensus exists on the proper terminology (i.e., definitions and distinctions) for meals and snacks (12). The term “snacking” is often used to describe eating behaviors that occur between “traditional” US mealtimes (i.e., breakfast, lunch, dinner); yet, many definitions of snacking vary according to energy contribution (4, 6), time of day of intake (3, 5, 13–15), and type of food or whether the consumer labels the eating occasion as a snack (5, 10, 16–19). As such, conflicting evidence exists in the literature as to whether snack consumption should be encouraged or restricted, and the contribution of snacks to the types and amounts of foods and beverages ingested remains largely unknown (18–23).

Previous iterations of the US Dietary Guidelines for Americans (DGA) have not provided any recommendations on eating frequency, although the DGA have consistently encouraged nutrient-dense food choices at breakfast and during snacking occasions for improved health outcomes (1, 24). Heightened public awareness and additional research efforts focused on specific aspects of eating frequency, including “grazing,” intermittent fasting, time-restricted eating, meal skipping, and late-night eating, suggest an increased level of interest in the evolving science of eating frequency. With an emerging scientific evidence base, an examination of eating behaviors and meal frequency with health outcomes across diverse populations is possible. Accordingly, an examination of whether eating frequency affects diet quality and health is warranted and was therefore addressed by the 2020 Dietary Guidelines Advisory Committee (DGAC) for the first time.

The objective of this paper is to provide a summary of the data examined and findings by the DGAC related to the types of eating occasions (i.e., meals and snacks) and the macronutrient contributions they provide, respectively, as well as the frequency of meal occasions relative to dietary quality among the US population (≥2 y), with a focus on sex, age, race and Hispanic origin, and family income, using nationally representative data from the NHANES. As such, portions of this work were previously published in a formal report to the US government (25).

## Methods

The 2020 DGAC comprised 20 non-federal scientists who assembled as a panel under the endorsement of the Federal Advisory Committee Act; the DGAC also received additional support from federal staff in the statistical review and analysis of data on eating frequency and the macronutrient contributions provided among the US population (25). The analysis submitted by the Federal Data Analysis Team was completed using What We Eat in America (WWEIA) data, the dietary component of NHANES 2013–2016.

### NHANES survey description

NHANES is a nationally representative, cross-sectional survey designed to assess the health and nutritional status of the noninstitutionalized, civilian residents of the United States, and is conducted by the National Center for Health Statistics (NCHS), CDC (26). NHANES uses a complex, stratified, multistage probability cluster sampling design. Data are collected and released for each 2-y cycle on the NHANES website (27, 28).

In NHANES, data on health and demographic information were first collected as part of an in-home interview, followed by an examination completed in person in the Mobile Examination Center (MEC). During the MEC visit, trained dietary interviewers collected information from participants via a 24-h dietary recall (24HR) using the USDA's Automated Multiple-Pass Method (AMPM) (27–29). The research ethics review board at the NCHS authorized all NHANES protocols; each participant (or proxy) provided written informed consent prior to study initiation. Details regarding NHANES dietary assessment can be found online (30).

For the current analyses, data available from 2 recent survey cycles (i.e., 2013–2014 and 2015–2016) were used. The responses rates for the NHANES 2013–2014 and 2015–2016 survey cycles were 68.4% and 62.3%, respectively. For analyses assessing overall diet quality [via the Healthy Eating Index (HEI)–2015], NHANES 2013–2014 and 2015–2016 were combined to obtain estimates with increased precision necessary for a Healthy Eating Index analysis. All other analyses were based on data from the NHANES 2015–2016 survey cycle. The analytic samples included Americans aged 2 y and older who had at least 1 reliable 24HR for NHANES 2015–2016 (analytic sample 1; *n* = 7918) and NHANES 2013–2016 (analytic sample 2; *n* = 15,985). Thus, for both analytic samples, those who were <2 y of age (*n* = 464, NHANES 2013–2014; *n* = 409 NHANES 2015–2016), did not participate in the day 1 24HR (*n* = 362, NHANES 2013–2014; *n* = 427, NHANES 2015–2016), or had unreliable or incomplete 24HR data were excluded (*n* = 1282, NHANES 2013–2014; *n* = 1217, NHANES 2015–2016).

### NHANES demographic and health-related characteristics

Demographic and health-related characteristics, including data on sex, age, race and Hispanic origin, and family income, were self-reported by participants during the household interview. Age (in years) was categorized as follows: 2–5 y, 6–11 y, 12–19 y, 20–29 y, 30–39 y, 40–49 y, 50–59 y, 60–69 y, and 70+ y; data were also examined among children and adolescents (ages 2–19 y) and adults (ages ≥20 y). Race and Hispanic origin groupings were categorized according to NCHS guidelines: non-Hispanic White, non-Hispanic Black, non-Hispanic Asian, and Hispanic. Family income was identified in accordance with the family poverty-to-income ratio (PIR), which examines family income in relation to family size (29, 31, 32). PIR is a commonly used indicator of family income and serves as a measure of income eligibility for federal nutrition-assistance programs, such as the Supplemental Nutrition Assistance Program (SNAP). Most notably, a PIR ≤130% indicates potential eligibility for SNAP benefits (33). Conversely, previous studies have utilized a PIR >350% to designate high-income families (34, 35). Accordingly, for the purposes of this analysis, 3 PIR categories were established: ≤130% (low income), 131–350% (middle income), and >350% (high income).

### NHANES dietary assessment

Dietary intake was self-reported via the in-person, day 1 24HR, conducted by trained interviewers in the MEC. Participants recalled all foods and beverages consumed over the prior 24-h period, which was recorded by trained interviewers using the USDA AMPM method (36). Dietary 24HRs were reported during the MEC examination by participants or their proxies depending on the age of the participant: children 2 to 5 y used a proxy, children 6 to 8 y used a proxy and assisted the proxy, older children 9 to 11 y self-reported the 24HR with the assistance of a proxy, and participants aged 12 y and older self-reported the 24HR. The USDA's Food and Nutrient Database for Dietary Studies, updated for the 2015–2016 NHANES cycle (37), was used to calculate the energy and macronutrient content of foods and beverages reported. For each eating occasion recalled on the 24HR, a time stamp, name of eating occasion, and all foods and beverages (including water) consumed during that eating occasion were recorded. Survey respondents selected the name of all eating occasions from a fixed list that was provided during the interview. According to the NHANES protocol, types of eating occasions consisted of breakfast, lunch, dinner, supper, brunch, snack, drink, and extended consumption (in addition to their Spanish equivalents: *desayano*, *almuerzo*, *comida*, *merienda*, *cena*, *entre comida*, *botana*, *bocadillo*, *tentempie, bebida*) (38, 39).

### Eating occasion definitions

For the purposes of this study, an eating occasion was defined as “any ingestive event (food or beverage, including water) that is either energy yielding or non-energy yielding,” whereas frequency of eating was defined as “the number of daily eating occasions.” All eating occasions were further divided into discrete meal categories (i.e., breakfast, lunch, dinner, and Spanish-language equivalents) and snacks (i.e., self-defined snacks, beverage only, and extended consumption events). Breakfast included all eating occasions designated by the respondent as “breakfast” or the Spanish equivalents *desayuno* and *almuerzo*; lunch included all eating occasions designated as “brunch,” “lunch,” or the Spanish equivalent *comida*; and dinner included all eating occasions designated as “dinner,” “supper,” or the Spanish equivalent *cena* (40). Snacks were reported as distinct eating occasions and consisted of 1 or more food and beverage items, including plain water. All reports of “snack,” “drink,” or “extended consumption” were included as snacks, as well as the Spanish equivalents *merienda*, *entre comida*, *botana*, *bocadillo*, *tentempie*, and *bebida*. Extended consumption was defined as an eating occasion that does not have a distinct period of consumption (e.g., sipping a cup of coffee throughout the day), and where the respondent can more easily report on the total amount consumed rather than smaller amounts throughout the day.

### Diet quality indicator

The HEI-2015 score was utilized to assess dietary quality (41). The HEI-2015 is a diet quality construct designed to measure how closely the dietary patterns of Americans adhere to the 2015–2020 DGA (1, 41, 42). The HEI-2015 is composed of 13 total dietary components; 9 of these components focus on food group and nutrient adequacy and include total fruit, whole fruit, total vegetables, greens and beans, whole grains, dairy, total protein foods, seafood and plant proteins, and fatty acids (41, 42). The other 4 components are refined grains, sodium, added sugars, and saturated fats, all of which are nutrient and/or food components that should be consumed in moderation (41, 42). The HEI-2015 score ranges from 0 to 100, with a greater score indicating higher diet quality. The HEI-2015 uses a density-based approach, meaning that it takes into consideration the amounts of food groups and nutrients consumed relative to energy intake, as opposed to the absolute quantity of foods and nutrients consumed (41, 42). It is also important to note that the HEI-2015 does not take into consideration any nutrient exposures from dietary supplements.

### Statistical analysis

Distributions of meal and snack patterns and estimated proportions of daily energy and macronutrient intakes from meals and snacks among the US population and population subgroups were computed using descriptive statistics.

HEI-2015 component and total scores were estimated using day 1 24HR data and the population ratio method; the HEI-2015 population ratio method has been previously described elsewhere (43), and was computed using publicly available SAS® macros distributed by the National Cancer Institute (43). Briefly, the population ratio method sums the intake of energy and the relevant food group and nutrient components for all individuals in a population to obtain population-level intake estimates. The ratio of each food group and/or nutrient component to energy is then computed and scored; the total score reflects the sum of the 13 component scores.

All statistical analyses were performed using SAS® version 9.4 (2012; SAS Institute, Inc.) and SAS®/STAT 14.2 (2016; SAS Institute, Inc.). SAS-callable SUDAAN®, release 11.0 (2012; RTI International), was used to account for the WWEIA, NHANES sample design in standard error estimation and statistical testing. NHANES dietary day 1 sample weights were used to adjust for oversampling, post-stratification, nonresponse, noncoverage, and differential probabilities of selection (27, 28). Day of the week of dietary recall was also adjusted for in the estimation process via NHANES dietary sampling weights (27, 28). For analyses assessing overall diet quality, estimates between groups were compared using a 2-sided *t* test with a Bonferroni correction for multiple comparisons; statistical significance was established at *P* < 0.0002. NCHS presentation standards were used for flagging unreliable estimates (44).

## Results

The majority of Americans (≥2 y) reported consuming 2 (31%) or 3 (64%) meals on a given day and 2 (23%) or 3 (22%) snacks on that day (**Tables 1** and **2**). More than one-third of the population consumed 3–4 snacks on a given day. However, the frequency of eating, specifically meal and snack patterns, varied by age, race and Hispanic origin, and family income. Most notably, the likelihood of reporting 3 meals on a given day varied by age. US adults (≥ 20 y) were less likely to report 3 meals on a given day, when compared with their child or adolescent counterparts (Table 1), and they reported the largest percentage of their daily energy intake at dinner time (36%), as compared with breakfast (18%), lunch (25%), and snacks (22%) (**Supplemental Table 1**). Among children (2–19 y), total daily energy was distributed among breakfast (18%), lunch (27%), dinner (32%), as well as snacks (23%) in a similar fashion to adults (Supplemental Table 1 and **Supplemental Table 2**).

**TABLE 1:** Distribution of meal patterns on a given day among the US population (≥2 y), by sex and age: NHANES 2015–2016^1^

	Meal patterns		
	Breakfast, lunch, and dinner^2^	Any 2 meals	Any 1 meal or less
Males			
Age group			
2–5 y	84 (2.1)	15 (1.9)	1* (0.3)
6–11 y	73 (3.7)	24 (3.4)	3* (1.0)
12–19 y	52 (2.7)	37 (1.6)	11 (1.9)
20–29 y	54 (4.1)	36 (3.4)	10 (2.1)
30–39 y	60 (2.9)	33 (2.6)	7 (1.4)
40–49 y	65 (4.3)	30 (3.2)	6 (1.9)
50–59 y	60 (3.5)	35 (4.0)	5 (1.4)
60–69 y	67 (5.7)	30 (5.4)	3* (0.7)
≥70 y	68 (2.2)	30 (2.2)	2* (0.8)
Children (2–19 y)	66 (2.7)	28 (1.9)	6 (1.1)
Adults (≥20 y)	61 (1.7)	33 (1.5)	6 (0.7)
All (≥2 y)	63 (1.7)	31 (1.2)	6 (0.7)
Females			
Age group			
2–5 y	89 (2.3)	11 (2.3)	^#^
6–11 y	72 (2.4)	24 (2.9)	4 (0.9)
12–19 y	49 (1.6)	41 (2.4)	10 (2.3)
20–29 y	59 (2.5)	33 (2.8)	7 (1.2)
30–39 y	66 (2.6)	29 (2.9)	5 (0.9)
40–49 y	70 (3.7)	27 (3.4)	4* (0.7)
50–59 y	65 (3.3)	32 (3.5)	2* (0.8)
60–69 y	66 (4.5)	30 (4.8)	4* (1.3)
≥70 y	68 (3.1)	28 (2.9)	4* (1.1)
Children (2–19 y)	65 (1.8)	29 (2.0)	6 (1.1)
Adults (≥20 y)	66 (1.6)	30 (1.7)	4 (0.4)
All (≥2 y)	65 (1.4)	30 (1.4)	5 (0.4)
Males and females			
Children (2–19 y)	66 (2.0)	28 (1.6)	6 (0.9)
Adults (≥20 y)	64 (1.5)	31 (1.3)	5 (0.4)
All (≥2 y)	64 (1.4)	31 (1.1)	5 (0.5)

1 Values are % (SE). *Indicates an estimate that does not meet National Center for Health Statistics standards of reliability. ^#^Indicates a non-zero value too small to report.

2 All eating occasions designated by the respondent as “breakfast,” “lunch,” and “dinner,” respectively, or their Spanish equivalents.

**TABLE 2:** Distribution of snack occasions among the US population (≥2 y), by sex, age, race and Hispanic origin, and family poverty-to-income ratio: NHANES 2015–2016^1^

	Snack occasions^2^							
	0	1	2	3	4	5	6	≥7
Males								
Children (2–19 y)	8 (1.2)	20 (1.4)	25 (1.7)	21 (1.4)	16 (1.3)	6 (0.7)	2 (0.4)	2 (0.5)
Adults (≥20 y)	7 (0.6)	17 (1.5)	22 (1.2)	20 (1.2)	16 (1.4)	8 (1.1)	5 (1.0)	3 (0.6)
All (≥2 y)	8 (0.7)	18 (1.2)	23 (0.9)	20 (1.0)	16 (1.2)	8 (0.9)	4 (0.8)	3 (0.5)
Females								
Children (2–19 y)	9 (1.1)	19 (2.3)	25 (1.3)	21 (1.3)	14 (1.8)	7 (1.0)	3 (0.5)	2 (0.4)
Adults (≥20 y)	5 (0.6)	17 (1.3)	23 (1.4)	25 (1.6)	14 (1.0)	9 (0.9)	3 (0.6)	5 (0.9)
All (≥2 y)	6 (0.5)	17 (1.3)	24 (1.2)	24 (1.2)	14 (0.8)	8 (0.8)	3 (0.4)	4 (0.8)
Males and females								
Children (2–19 y)	9 (1.0)	20 (1.5)	25 (0.9)	21 (1.2)	15 (1.1)	6 (0.6)	2 (0.3)	2 (0.3)
Adults (≥20 y)	6 (0.4)	17 (1.1)	23 (1.2)	23 (1.2)	15 (0.9)	8 (0.6)	4 (0.6)	4 (0.6)
All (≥2 y)	7 (0.5)	18 (1.1)	23 (0.8)	22 (0.9)	15 (0.8)	8 (0.6)	4 (0.5)	4 (0.5)
Non-Hispanic White	5 (0.4)	15 (1.2)	22 (1.0)	24 (1.2)	16 (1.1)	9 (0.7)	4 (0.7)	4 (0.6)
Non-Hispanic Black	10 (1.0)	23 (1.3)	27 (1.4)	19 (1.1)	12 (0.8)	5 (0.8)	2 (0.5)	2 (0.3)
Non-Hispanic Asian	7 (1.2)	17 (1.5)	23 (1.3)	19 (1.6)	16 (1.2)	8 (1.1)	4 (1.1)	6 (1.3)
Hispanic	10 (1.3)	22 (1.4)	25 (1.2)	20 (1.2)	12 (1.0)	6 (0.9)	3 (0.5)	2 (0.4)
<131% PIR	11 (0.9)	22 (1.5)	25 (1.3)	21 (1.0)	12 (1.0)	5 (0.7)	2 (0.3)	2 (0.4)
131–350% PIR	7 (0.7)	19 (1.3)	25 (0.9)	22 (1.1)	14 (0.9)	7 (0.8)	4 (0.7)	3 (0.5)
>350% PIR	4 (0.4)	14 (1.3)	20 (1.7)	24 (1.7)	18 (1.3)	10 (0.8)	4 (0.8)	5 (0.9)

1 Values are % (SE). PIR, family poverty-to-income ratio.

2 Snack occasions were reported as distinct eating occasions during the dietary interview and consisted of ≥1 food and beverage items, including plain water. All reports of “snack,” “drink,” or “extended consumption” (items that were consumed over a long period of time) were included as snack occasions. Spanish-language interviewers used Spanish-language snack occasion names: *merienda*, *entre comida*, *botana*, *bocadillo*, *tentempie*, and *bebida*.

Comparable patterns were also observed when evaluating differences in eating behaviors by race and Hispanic origin. The frequency of meals and snacks varied by race and Hispanic origin, with Hispanic and non-Hispanic Black Americans reporting lower consumption of 3 meals (i.e., breakfast, lunch, and dinner) on a given day as compared with other race and Hispanic origin groups (**Table 3**). Additionally, Hispanic children and adults tended to consume a higher amount of energy and macronutrients as a percentage of daily intake at breakfast than any other race or Hispanic origin group (Supplemental Table 1 and **Supplemental Table 3**).

**TABLE 3:** Distribution of meal patterns on a given day among the US population (≥2 y), by race and Hispanic origin: NHANES 2015–2016^1^

	Meal patterns		
	Breakfast, lunch, and dinner^2^	Any 2 meals	Any 1 meal or less
Non-Hispanic White			
Adults (≥20 y)	68 (1.8)	28 (1.7)	3 (0.4)
All (≥2 y)	69 (1.7)	27 (1.5)	4 (0.4)
Non-Hispanic Black			
Adults (≥20 y)	49 (1.9)	37 (1.6)	14 (1.1)
All (≥2 y)	52 (1.7)	35 (1.6)	12 (1.0)
Non-Hispanic Asian			
Adults (≥20 y)	73 (3.3)	24 (2.6)	3* (1.1)
All (≥2 y)	74 (3.0)	23 (2.4)	3 (0.9)
Hispanic			
Adults (≥20 y)	49 (2.4)	44 (2.2)	7 (1.1)
All (≥2 y)	51 (1.7)	42 (1.5)	7 (0.9)

1 Values are % (SE). *Indicates an estimate that does not meet National Center for Health Statistics standards of reliability.

2 All eating occasions designated by the respondent as “breakfast,” “lunch,” and “dinner,” respectively, or their Spanish equivalents.

Differences were also observed when assessing the frequency of meals by family income. Those whose family income was less than 131% of the federal poverty level were more likely to report the lowest number of meal occasions (i.e., ≤1 meal) when compared with those who had a family income greater than 131% of the poverty level (**Table 4**). Individuals with the lowest income (PIR <131%) had a slightly lower proportion of macronutrients consumed at lunch when compared with other income groups (Supplemental Table 3).

**TABLE 4:** Distribution of meal patterns on a given day among the US population (≥2 y), by family poverty-to-income ratio: NHANES 2015–2016^1^

	Meal patterns		
	Breakfast, lunch, and dinner^2^	Any 2 meals	Any 1 meal or less
<131% PIR			
Adults (≥20 y)	50 (2.5)	39 (2.4)	11 (1.1)
All (≥2 y)	53 (1.9)	36 (1.4)	10 (1.0)
131–350% PIR			
Adults (≥20 y)	61 (1.7)	34 (1.4)	5 (0.6)
All (≥2 y)	62 (1.6)	33 (1.2)	5 (0.6)
>350% PIR			
Adults (≥20 y)	74 (2.1)	24 (1.9)	2 (0.5)
All (≥2 y)	73 (1.9)	24 (1.7)	2 (0.5)

1 Values are % (SE). PIR, family poverty-to-income ratio.

2 All eating occasions designated by the respondent as “breakfast,” “lunch,” and “dinner,” respectively, or their Spanish equivalents.

As noted above, snacking was ubiquitous in the US population (93%) (Table 2 and Supplemental Table 2). While these types of eating occasions most often occur in the afternoon between lunch and dinner, they also provide nearly 22% of total daily energy intake for most Americans (Supplemental Table 2).

Differential patterns of snacking occurred by family income level. Across all ages, those whose family income was less than 131% of the poverty level were the least likely to report consuming meals (i.e., breakfast, lunch, dinner) or snacks, when compared with their higher income counterparts (i.e., PIR 131–350% and PIR >350%) (Supplemental Tables 1 and 2).

When evaluating the relation between meal patterns and dietary quality using the HEI-2015, Americans who consumed 3 meals on a given day (HEI-2015 score = 61) consistently reported a diet higher in dietary quality than Americans who consumed 2 meals on a given day (HEI-2015 score = 55) (*P* < 0.001; **Table 5**). These findings were also consistent with individual HEI-2015 dietary quality components. Americans who consumed 3 meals on a given day were likely to have higher diet quality as a result of higher intakes of several adequacy components, including total vegetables, greens and beans, total fruit, whole fruit, whole grains, and dairy (all *P* < 0.001) and lower intakes of some moderation components, such as added sugar and sodium (both *P* < 0.001), than those who consumed only 2 meals on a given day (Table 5).

**TABLE 5:** HEI-2015 total and component scores on a given day among the US population (≥2 y), by the number of meals consumed: NHANES 2013–2016^1^

	Breakfast, lunch, and dinner	Any 2 meals	Difference in means	*P* (α = 0.0002)
HEI-2015 component				
Total vegetables	3.48 (0.11)	3.17 (0.08)	0.308	<0.001
Greens and beans	3.34 (0.24)	2.77 (0.18)	0.568	<0.001
Total fruit	3.16 (0.12)	2.36 (0.13)	0.807	<0.001
Whole fruit	4.70 (0.22)	3.18 (0.19)	1.518	<0.001
Whole grains	3.35 (0.15)	2.32 (0.13)	1.031	<0.001
Dairy	6.32 (0.18)	5.43 (0.16)	0.898	<0.001
Total protein foods	5.00 (0.00)	5.00 (0.00)	0.000	—
Seafood and plant protein	5.00 (0.00)	4.87 (0.17)	0.134	<0.001
Fatty acid ratio	4.14 (0.21)	4.10 (0.12)	0.045	<0.001
Sodium	3.30 (0.15)	4.42 (0.26)	−1.125	<0.001
Refined grains	6.40 (0.15)	6.38 (0.20)	0.015	0.0005
Saturated fat	5.05 (0.16)	5.29 (0.17)	−0.245	<0.001
Added sugar	7.34 (0.14)	6.04 (0.20)	1.305	<0.001
HEI-2015 total score	60.6 (1.0)	55.3 (0.9)	5.26	<0.001

1 Values are HEI score (SE) unless otherwise indicated. HEI, Healthy Eating Index.

## Discussion

Eating behaviors are most commonly characterized by the frequency, types, and amounts of foods ingested. The frequency of eating is associated with a wide range of appetitive, digestive, and metabolic processes that are relevant to the health and well-being of Americans (7, 45–50). While the types and amounts of foods consumed are a traditional focus of the US DGA and DGAC, the 2020 Committee was the first to directly address the question of frequency of eating. Understanding the frequency of eating in the US population is critical, given that changes in the frequency of eating may be associated with changes in a person's overall nutritional and health status (7, 46, 49–52).

Traditionally, eating occasions aligned closely with the conventional naming of meals, such as breakfast (e.g., 07:00 to 09:00), lunch (e.g., 12:00 to 13:00), and dinner (e.g., 18:00 to 20:00). While conventional naming of meals helps shape the context for providing and tailoring recommendations, the frequency of conventional meals no longer reflects the dietary practices of the majority of the US population. Most Americans now report consumption of approximately 5.7 daily eating occasions, inclusive of meals, snacks, beverages, and extended consumption, that occur most often at noon or in the “evening.” These daily eating occasions are primarily composed of 2 to 3 meals and 2 to 3 snacks on a given day. Ninety-three percent of the US population reported at least 1 snack on a given day, which is often consumed in the afternoon between lunch and dinner. According to the American Time of Use Survey (ATUS), increased constraints on time are resulting in a larger proportion of Americans participating in eating occasions on the go or while working (11), which may contribute to the shift in the American dietary patterns towards secondary eating occasions. This trend raises health questions as the highest diet quality was observed among Americans with a more traditional dietary pattern (i.e., consumption of 3 meals on a given day). The ATUS study population was also most likely to consume 2 to 3 snacks on a given day (as opposed to ≤1 snack or ≥4 snacks) (11), which is aligned with the number of snacks most commonly consumed among the general US population. However, the analysis of this nationally representative sample of the US population also indicates that the frequency of types of eating occasions is shaped by a number of demographic characteristics, such as age, race and Hispanic origin, and family income.

While the relation between eating frequency and the quality of the American diet has been investigated, few studies have examined separately the effects of meal frequency on dietary quality. An earlier study conducted by Murakami and Livingstone (53) concluded that higher meal and snack frequency resulted in higher HEI-2010 scores among US men and women, after accounting for the confounding effects of energy intake misreporting. The magnitude of the association was stronger for higher meal frequency when compared with higher snack frequency (53). Similar studies have also suggested that meal frequency, but not snack frequency, is positively associated with higher dietary quality among adolescents (54); yet, several findings also support the view that snacks have the potential to increase dietary quality among children (54). Snacks alone provide as much as 35% of total added sugars among children (25). Thus, snacks are a popular target for management of energy intake. However, snacks also have the potential to contribute to diet acceptability, quality of life, and increased macronutrient content (55). Thus, conscious, healthful snack choices among US children may aid in improving their dietary quality (56). Alternative eating practices (i.e., reducing snacking or improving the nutrient density and reducing the energy density of snacks) may also augment diet quality among children (16, 57, 58), adolescents (17), and adults (21).

### Research needs

While data characterizing eating frequency and dietary patterns exist at the national level, much fewer data are available to examine the relations between frequency of eating and health. Indeed, the DGAC was tasked with evaluating a systematic review of literature on frequency of eating and various health outcomes across the life course. How frequency of eating is defined and examined is not consistent, making synthesis of the available literature challenging. Due to the inconsistency and limitations in the body of evidence included in the systematic review, the evidence was deemed insufficient to draw conclusions about the relations between frequency of eating and growth, size, body composition, and risk of overweight and obesity, cardiovascular disease, and type 2 diabetes. Thus, a research need exists to standardize frequency of eating terms and ingestive events across study designs, and to complete more well-designed, randomized controlled trials that directly examine whether a causal role exists between frequency of eating and health. Future studies on frequency of eating should consider including all the necessary data in a study to assess frequency of eating and outcomes, including key confounders and adequate dietary data collection.

Additionally, intermittent fasting, intermittent energy restriction, time-restricted eating, breakfast skipping, and late-night eating are all topics of current public interest. The manipulation of eating frequency and timing of ingestion is at the core of each of these eating behaviors. Although timing of eating occasions is also an important consideration in the relation between frequency of eating and health, the number and timing of daily eating occasions could be evaluated as the primary intervention/exposure or exposure of interest and health. Given the differences observed in timing and frequency of eating varied by key sociodemographic factors like family income in the United States, future research could address how food insecurity and other constraints on food choice and access may relate to health.

### Strengths and limitations

This study analyzed data from the NHANES, a nationally representative sample of the US noninstitutionalized population. The findings are limited by the cross-sectional nature of the data and, therefore, do not allow inferences regarding temporality or causation. Limitations, such as energy underreporting, have been previously associated with self-report dietary data (e.g., 24HRs) and are well-characterized in the literature (30, 59, 60). Previous studies have shown that energy misreporting varies with different eating occasions (e.g., energy underreporting is more prevalent with snacks when compared with meals) (61, 62). However, self-report dietary data can be useful for examining frequency-of-eating data at the population level and are important for monitoring the health and nutritional status of the population over time (59, 60).

### Conclusions

Taken together, these findings support the recommendation to make nutrient-dense food choices at most eating occasions. Healthy dietary patterns can be constructed in a variety of ways to suit differing life stages, cultural practices, and income levels; continued efforts to improve diet quality and careful planning of nutrient-dense meals are presently advisable (25).

## Data Availability

Data described in the manuscript are publicly available without restriction at: https://wwwn.cdc.gov/nchs/nhanes/default.aspx. Data analysis was performed by the Federal Data Analysis Team for the Dietary Guidelines Advisory Committee and inquiries regarding the codebook and/or analytic code should be made to them.

## Notes

The authors report no funding received for this study.

The authors do not have any conflicts of interest to disclose. The findings and conclusions in the present manuscript represent those of the authors and do not reflect the views of the U.S. government or their corresponding universities. This work was conducted under the Federal Advisory Committee Act.

Supplemental Tables 1–3 are available from the “Supplementary data” link in the online posting of the article and from the same link in the online table of contents at https://academic.oup.com/cdn/.

Abbreviations used: AMPM, Automated Multiple-Pass Method; ATUS, American Time of Use Survey; DGA, Dietary Guidelines for Americans; DGAC, Dietary Guidelines Advisory Committee; HEI, Healthy Eating Index; MEC, Mobile Examination Center; NCHS, National Center for Health Statistics; PIR, poverty-to-income ratio; SNAP, Supplemental Nutrition Assistance Program; WWEIA, What We Eat in America; 24HR, 24-hour dietary recall.
